# Effect of intrauterine administration of human chorionic gonadotropin one day before fresh blastocyst transfer on clinical outcomes: a quasi-experimental study

**DOI:** 10.11604/pamj.2022.42.27.31539

**Published:** 2022-05-12

**Authors:** Henda Mustapha, Marwa Lahimer, Mehdi Makni, Imene Bannour, Ons Kaabia, Mouna Derouich, Mohamed Aymen Ferjaoui, Ramzi Arfaoui, Monia Zaouali, Mounir Ajina

**Affiliations:** 1 Unit of Reproductive Medicine, Farhat Hached University Teaching Hospital, Sousse, Tunisia,; 2 Department of Obstetrics and Gynecology, Farhat Hached University Teaching Hospital, Sousse, Tunisia,; 3 Department B of Gynecologic Surgery, Maternity Center, Tunis, Tunisia,; 4 Department of Maternity, Military Hospital of Instruction, Tunis, Tunisia,; 5 Laboratory of Physiology and Functional Exploration, Faculty of Medicine, Sousse, Tunisia

**Keywords:** Human chorionic gonadotropin (hCG), intrauterine administration, blastocyst transfer, implantation, IVF, clinical outcomes

## Abstract

**Introduction:**

embryo implantation is a crucial step for assisted reproductive technology (ART) achievement. Human chorionic gonadotropin (hCG) is one of the main regulators of the implantation process. Studies focusing on the impact of intrauterine hCG infusion at the time of embryo transfer on clinical ART outcomes have shown controversial results, mainly at blastocyst stage. In this study, we aimed to investigate whether intrauterine hCG infusion one day before human blastocyst transfer in fresh invitro fertilization (IVF) cycles enhances implantation and pregnancy rates.

**Methods:**

a total of 174 subfertile women undergoing autologous fresh blastocyst transfer were enrolled in this randomized prospective study. Patients were randomly divided into three groups; group 1 (n = 54) and group 2 (n = 59) received an intrauterine injection of respectively 500 IU and 1000 IU of hCG one day before blastocyst transfer and the control group (n= 61) did not receive any intrauterine injection. The pregnancy and implantation rates were compared between the three study groups.

**Results:**

significant difference was found between the study groups. The bio chemical pregnancy rates were 25.9%, 30.5% and 29.5%, the clinical pregnancy rates were 24.1%, 27.1% and 27.9% and the implantation rates were 14.9%, 17.9% and 18.7% respectively in group 1,2 and control group.

**Conclusion:**

our results have shown that clinical outcomes in fresh IVF cycles cannot be improved through intrauterine hCG administration one day prior to blastocyst transfer, neither with 500 IU of hCG nor with a higher dose of 1000 IU of hCG.

## Introduction

In recent years, despite major breakthroughs achieved in the ART field, the pregnancy and delivery rates remain relatively low in subfertile couples attempting to conceive using these techniques. Embryo implantation is considered as the major obstacle limiting the success of IVF-embryo transfer (ET) cycles. It is estimated that approximately 50-75% of pregnancy losses are caused by implantation failure [1,2]. Implantation is an extremely complex and well-coordinated process, requiring high-quality embryos and a receptive endometrium [3]. Fundamentally, successful implantation depends on synchronized embryo-maternal dialogue mediated by many factors, including ovarian steroid hormones, local endometrium autocrine and paracrine signaling, and embryo-derived signals [4,5]. Human chorionic gonadotropin is the first known human embryo derived signal, believed to be a key regulator during the implantation process. It is implicated in several molecular pathways that promote trophoblast invasion, modulate immunological tolerance and stimulate endometrial angiogenesis at the maternal-fetal interface, essential conditions for pregnancy progression [6,7].

The hCG subunits are one of the earliest molecules secreted by cleavage stage embryos and later produced in higher concentrations at the blastocyst stage [8]. In vitro embryo culture, especially until the blastocyst stage decreases hCG signaling to the endometrium during the early days of embryonic development, which may contribute to the relatively low implantation rate in IVF cycles [9]. Accordingly, it was suggested that hCG administration in the uterine cavity during the embryo implantation window might be a useful approach aiming to enhance clinical outcomes after IVF-ET procedure. The first randomized clinical trial using intrauterine hCG infusion before ET was conducted in 2011 by Mansour *et al*. and showed a significant improvement of clinical pregnancy rate compared to controls using the optimal concentration of 500 IU of hCG [10]. Similar findings were described in several subsequent studies on cleavage stage ETs [11-14]. However, there were only few studies focusing on blastocyst transfer (BT) exclusively and yielded controversial results [3,9,15,16]. Furthermore, the majority of these studies used the dose of 500 IU of hCG based on the pilot study conducted by Mansour and collaborators. However, there is no information about the potential effects of a higher dose of 1000 IU at the blastocyst stage. Hence, the present study was designed to assess whether the intrauterine injection of hCG one day before fresh BT in IVF cycles can have a beneficial effect on pregnancy and implantation rates. A unique administration of either 500 IU or 1000 IU of hCG was tested and compared to the control group.

## Methods

**Study design and setting:** the study consisted in a randomized controlled trial comparing between two intervention groups (500 IU and 1000 IU of intrauterine hCG) and control group. It was conducted at the Reproductive Medicine Unit of Farhat Hached University Teaching Hospital (Sousse, Tunisia) between March 2017 and June 2018.

**Study population:** women undergoing fresh BT within IVF cycles, were enrolled in this study. Each patient was included in the study with a single IVF cycle. The defined inclusion criteria were female age in the range of 20-43 years and fresh autologous BT on day five. The exclusion criteria were defined as failure to have the proper endometrial thickness for ET (<8mm measured by transvaginal ultrasound), presence of intrauterine adhesion, endometrial polyps, or uterine submucosal myomas, adenomyosis or endometriosis of stage III or higher. The sample size was calculated using an online sample size calculator (ClinCalc.com). A significant increase in implantation rate was considered to be 20%, and with α = 5% and β = 20%, a total of 180 women were needed. Patients who were lost to follow-up or experienced ET cancellation were excluded from the analysis.

**Randomization and study groups:** eligible patients were randomly assigned to three study groups with a random number generator using the Statistical Package for Social Sciences (SPSS) software version 22.0 for Windows (IBM SPSS Statistics, 22) based on their registration number in order of referral. Group 1 received an intrauterine injection of 500 IU of purified-urinary hCG one day before BT, group 2 received 1000 IU of hCG and the control group did not receive any intrauterine injection.

**Ovarian stimulation:** all patients underwent controlled ovarian hyperstimulation (COH) using either a gonadotrophin-releasing hormone (GnRH) agonist, GnRH antagonist or mild stimulation protocol. The COH protocol and the initial dose of gonadotropin (Gonal-F®, Merck Serono, Europe Limited UK) were chosen based on the patient characteristics. Patients were monitored by transvaginal ultrasound and serum estradiol dosage every 2-4 days. The gonadotropin dose was adjusted during stimulation according to the patient individual ovarian response. When at least 2 follicles reached a mean diameter ≥ 17mm, 250µg of recombinant hCG (Ovitrelle® Merck Serono, Europe Limited UK) or 10,000 IU of urinary hCG (Diclair®-HP-HCG 5000, BBT Biotech GmbH Germany) was administered s.c for ovulation triggering. Oocyte pick-up was performed 34-36 hours later.

**Embryo culture:** depending on the patients´ medical history and male partner sperm parameters, oocytes were fertilized either by conventional IVF or intracytoplasmic sperm injection (ICSI). Embryo culture was performed in sequential media (G-1™ PLUS and G-2™ PLUS; Vitrolife, Sweden). On day 2 after oocyte retrieval, cleavage stage embryos were evaluated according to the consensus scoring system of Istanbul workshop of 2011 [17]. On day 5, blastocyst quality was morphologically assessed by a professional embryologist, and 1-2 of the best blastocysts were selected for intrauterine transfer. Morphological grading was performed according to the classification of Gardner and colleagues [18]. Blastocysts with 3-5 expansion degree and with trophectoderm and inner cell mass of grad A, B or a combination of A and B were classified as top quality blastocysts. All other blastocysts were classified as non-top blastocysts.

**Human chorionic gonadotropin administration:** intrauterine administration of hCG in the two treatment groups (1 and 2) was performed on day 4 after ovum pick-up (OPU). The reason for choosing one day before blastocyst transfer was that hCG infusion into the endometrial cavity on day 4 is more synchronous with the physiological timing of embryonic hCG secretion, which typically begins to increase at the morula stage. Furthermore, it allows extending hCG endometrial exposure time, but without causing detrimental effects on endometrial LH-hCG receptors (LHCGR). The hCG solution was prepared by dissolving 5000 IU of purified urinary hCG powder (Diclair®-HP-HCG 5000, BBT Biotech GmbH Germany) in 0,5mL or 1mL of physiological saline according to the allocation instructions. Then, 100μL of the final preparation containing 500 IU or 1000 IU hCG (respectively in group 1 and 2) was infused into the uterine cavity using an intrauterine insemination catheter (Prince Medical, Ellios BioTek; France).

**Embryo transfer:** blastocyst transfer was performed on day 5 according to our standard ET protocol. The patient was put in a lithotomy position and a spectrum was inserted to visualize the cervix. The cervical mucus and any vaginal discharge were removed with small sterile cotton swabs. Selected blastocysts were transferred using a soft embryo replacement catheter (Inventcath Eco for Embryo Transfer, CDD; France) 0.5cm below the uterine fundus under ultrasound guidance. After ET the catheter was checked for retained embryos under the microscope. All patients received luteal phase support with 800 mg/day of transvaginal progesterone suppositories (Cyclogest; Actavis UK limited) from the day of oocyte retrieval. Serum β-hCG levels were measured 14 days after ET. In case of positivity, transvaginal ultrasonography was performed 3-5 weeks later.

**Data collection:** patient medical information regarding their age, male partner age, basal hormonal profile, infertility type, duration and origin, ovarian stimulation characteristics (protocol, duration, endometrial thickness and estradiol level on day of ovulation triggering), IVF technique, biological and clinical IVF outcomes (number and maturation of retrieved oocytes, fertilization and segmentation rate, number and quality of obtained/transferred embryos, βhCG dosage and ultrasound visualization of gestational sac) were collected from their medical records.

**Outcome measures:** the main analyzed outcomes were the implantation rates, biochemical pregnancy rates and clinical pregnancy rates. Biochemical pregnancy was considered as positive serum βhCG (≤25 IU/L) measured two weeks after ET. However, clinical pregnancy was characterized by the presence of a gestational sac with a fetal heart-beat identified through transvaginal ultrasonography 4-5 weeks after ET. The biochemical and clinical pregnancies rates were calculated per the number of the transfer. The implantation rate was defined as the number of gestational sacs based on the number of transferred embryos.

**Statistical analysis:** the statistical analysis was performed using SPSS software version 22.0 (IBM SPSS Statistics, 22). Data are presented as means ± standard deviation (SD) for quantitative variables and as percentages (%) for categorical variables. There was no missing data for the main outcomes analyzed. Means of continuous variables were compared between different groups (500 IU hCG, 1000 IU hCG, and control) using a one-way analysis of variance test (ANOVA). Chi-square test (X^2^) or Fisher exact test was used for qualitative variables as appropriate. A p value ≤ 0.05 was considered as statistically significant. Patients who were lost to follow-up or experienced ET cancellation were excluded from the analysis.

**Ethical considerations:** the study was approved by the Ethics Committee of Farhat Hached Hospital and was conducted according to its guidelines. All enrolled patients were accurately informed about the study protocol and about the unknown effectiveness of hCG intrauterine injection in improving pregnancy outcomes. Patients had the choice to participate or with draw from the study at any given point. Written informed consent was obtained from all participants before enrollment into the study. Strict confidentiality of patient information was ensured when handling data during all processes of data collection, capturing, analysis and storage.

## Results

**Figure 1:**
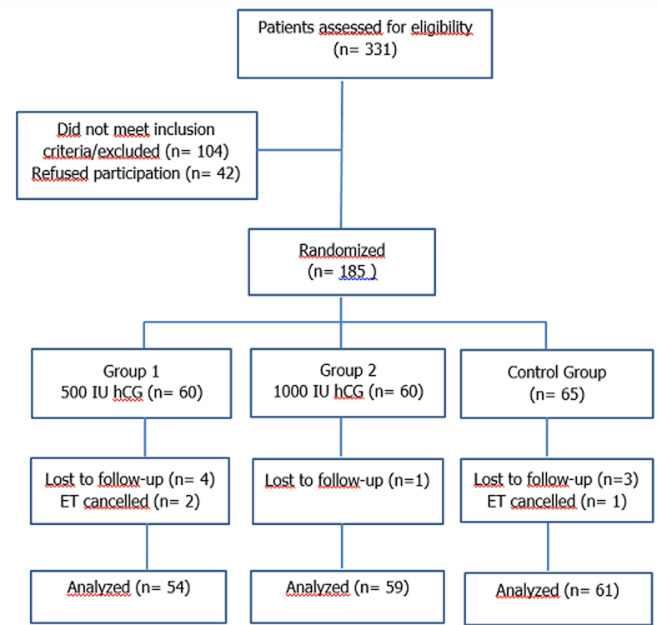
flow diagram of participants throughout the study

Out of 331 patients who were assessed for eligibility, 146 were excluded. Thus, 185 patients were randomized to three study groups, each consisting of 60 patients in the two HCG-treated groups and 65 patients in the control group. During the study, 4 patients out of group 1 (500 IU HCG), 1 out of group 2 (1000 IU HCG), and 3 patients out of the control group were lost to follow-up. Two patients in group 1 and one patient in the control group experienced ET cancellation due to ovarian hyper stimulation syndrome risk or fever episode (observed in only one patient in group 1). Thus, the final number of patients completing follow-up and analysis was 174 with 54, 59, and 61 patients respectively in groups 1, 2, and the control group. In Figure 1 represents the flow diagram of participants throughout the trial. All patients were followed for at least 6-7 weeks after ET. There was no missing data for the main outcomes analyzed. In this study, the hCG treatment was well tolerated by all patients and no side effects were observed in any one of the included patients. A total of 320 blastocysts were transferred to 174 patients.

The mean age of the study population was 32.85 ± 5.10 years. The baseline characteristics of the patients were comparable between the three study groups, as no significant differences were observed in terms of female and male age, infertility type, duration, and origin as well as basal hormonal levels for the female partner (Table 1). Likewise, the IVF technique and ovarian stimulation characteristics were similar between the three study groups, as shown in Table 2, with the exception of a significant difference observed in the estradiol level on the day of ovulation induction between group 1 and the control group. Moreover, the number of retrieved oocytes, the rates of oocytes maturation, fertilization, and segmentation were statistically comparable among the three study groups; as well as the total number and quality of day 2-3 obtained embryos (Table 3). The mean number of transferred blastocysts was of 1.87 ± 0.58 in group 1, compared to 1.90 ± 0.35 in group 2 and 1.75 ± 0.47 in control group (p = 0.217). The mean number of Top blastocyst transferred was of 0.80 ± 0.76, 1.00 ± 0.81 and 0.97 ± 0.81 respectively in group 1, 2 and control group (p= 0.345). The outcome's analysis revealed no evidence of difference with regard to biochemical pregnancy rate between the two hCG-treated groups and the control group; with respectively 25.9%; 30.5% and 29.5% (p = 0.854). In addition, there was no significant difference in terms of clinical pregnancy rate among the three groups; with 24.1%, 27.1%, and 27.8% respectively in groups 1, 2 and the control group (p= 0.890). Similarly, the embryo implantation rate was statistically comparable between all groups with 14.9% in group 1, 17.9% in group 2, and 18.7% in the control group (p= 0.744) (Table 3).

**Table 1:** baseline demographic and clinical characteristics of patients among the three study groups

		Group 1 500 IU hCG (n= 54)	Group 2 1000 IU hCG (n= 59)	Control group (n= 61)	Intergroup comparisons: p value
**Age (years)**	Women	34.58 ± 5.66	32.25 ± 5.06	32.54 ± 4.73	0.101
	Men	40.10 ± 6.32	38.26 ± 5.55	38.69 ± 5.69	0.349
**Day 3 hormonal assessment for female partners**	FSH (UI/l) LH (UI/l) AMH (ng/ml) E2 (pg/ml)	7.46 ±3.81 4.39 ±3.09 3.27 ±2.24 53.42 ±21.67	7.87 ± 2.32 5.12 ± 2.59 2.96 ± 2.91 58.85 ±30.33	7.39 ± 2.46 6.13 ± 3.93 3.77 ± 3.24 53.20 ±30.51	0.634 0.07 0.365 0.614
**Infertility origin**	Male (%) Female (%) Both (%) Idiopathic (%)	26 (48.1 %) 14 (25.9 %) 7 (13 %) 7 (13 %)	37 (62.7 %) 6 (10.2 %) 10 (16.9 %) 6 (10.2 %)	36 (59 %) 10 (16.4 %) 10 (16.4 %) 5 (8.2 %)	0.402
**Infertility duration (years)**		5.58 ±3.11	4.95 ± 2.88	4.83 ± 2.91	0.485
**Infertility type**	Primary secondary	44 (81.5 %) 10 (18.5 %)	47 (79.7 %) 12 (20.3 %)	55 (90.2 %) 6 (9.8 %)	0.248

FSH: follicle-stimulating hormone; LH: luteinizing hormone; AMH: anti-müllerian hormone; E2: estradiol; * statistically significant difference (p<0.05); data presented as mean ± SD or n (%)

**Table 2:** invitro fertilization cycle and ovarian stimulation characteristics among the three study groups

		Group 1 500 IU hCG (n= 54)	Group 2 1000 IU hCG (n= (59)	Control group (n= 61)	Intergroup comparisons p value
**IVF technique**	ICSI	33 (61.1 %)	39 (66.1%)	45 (73.8%)	0.657
	cIVF	17 (31.5 %)	15 (25.4%)	13 (21.3%)	
	Half-ICSI	4 (7.4 %)	5 (8.5%)	3 (4.9%)	
**Ovarian stimulation protocol**	Antagonist	23 (42.6 %)	23 (39%)	27 (44.3%)	0.320
	Long agonist	16 (29.6%)	15 (25.4%)	22 (36.1%)	
	Short agonist	11 (20.4 %)	17 (28.8%)	12 (19.7%)	
	Mild stimulation	4 (7.4%)	4 (6.8%)	0 (0%)	
Endometrial thickness on day of ovulation triggering (mm)		10.06 ± 2.34	9.88 ± 2.97	9.92 ± 1.59	0.944
E2 on the day of ovulation triggering (UI/l)		1634.40 ± 809.61	1867.06 ± 972.75	2233.65 ± 1175.13	0.02*
Ovarian stimulation duration (days)		10.00 ± 2.19	9.62 ± 2.18	10.19 ± 1.83	0.373
Total gonadotrophin dose (UI)		1896.25 ±665.42	1989.66 ± 710.94	1971.52 ± 597.63	0.815

cIVF: conventional IVF ; ICSI: intracytoplasmic sperm injection ; E2: estradiol * statistically significant difference (p<0.05); data presented as mean ± SD or n (%)

**Table 3:** invitro fertilization cycle outcomes among the three study groups

		Group 1 500 IU hCG (n= 54)	Group 2 1000 IU hCG (n= 59)	Control group (n= 61)	Intergroup comparisons: p value
Number of retrieved oocytes		6.40 ± 3.78	7.91 ± 3.80	7.36 ± 4.04	0.230
Oocyte maturation rate (%)		89.56 ± 13.01	86.87 ±13.50	85.25 ± 14.71	0.430
Fertilization rate (%)		85.49 ± 18.87	80.87 ± 18.93	81.84 ± 18.16	0.489
Segmentation rate (%)		100 ± 0	97.98 ± 6.78	98.74 ±5.21	0.253
Number of day 2-3 obtained embryos (n)		4.43 ± 2.16	4.96 ± 2.04	4.84 ± 2.51	0.569
TOP quality cleavage stage embryo rate (%)		72.25 ± 20.54	69.20 ± 22.68	74.76 ±26.83	0.411
Number of transferred blastocyst		1.87 ± 0.58	1.90 ± 0.35	1.75 ± 0.47	0.217
Number of top blastocyst transferred		0.80 ± 0.76	1.00 ± 0.81	0.97 ± 0.81	0.345
Embryo transfer quality	Easy	50 (92.6 %)	56 (94.9 %)	55 (90.2 %)	0.613
	Difficult	4 (7.4 %)	3 (5.1 %)	6 (9.8 %)	
Biochemical pregnancy (%)		14/54 (25.9 %)	18/59 (30.5 %)	18/61 (29.5 %)	0.854
Clinical pregnancy (%)		13/54 (24.1 %)	16/59 (27.1 %)	17/61 (27.9 %)	0.890
Implantation rate (%)		15/101 (14.9%)	20/112 (17.9%)	20/107 (18.7%)	0.744

* statistically significant difference (p<0.05) data are presented as mean ± SD or n (%)

## Discussion

Human chorionic gonadotropinis a glycoprotein hormone with three identified heterodimeric isoforms resulting from post-translational modifications produced by different cells [19]. It is widely admitted that hCG is a key player in the complex process of embryo implantation, fulfilling a large spectrum of various biological functions. Among other actions, the main hCG molecule and its variants are chiefly involved in decidualization of the endometrial stromal cells, trophoblast invasion, the proliferation of uterine natural killer (uNK) cells, immunological modulation at the maternal-fœtal interface, stimulation of endometrial angiogenesis, and maintenance of progesterone secretion by the corpus luteum [6,7,19,20]. The multifaceted roles of hCG in endometrial receptivity led researchers to explore the clinical effect of intrauterine hCG infusion before ET on IVF outcomes. Many randomized controlled trials [3,9-16], meta-analysis [21,22], and reviews [5,23,24] were performed to assess the effectiveness and the relevance of this approach in various settings. It should be noted that the majority of studies were related to cleavage stage embryos and only a few studies focused exclusively on BT and yielded inconsistent and conflicting findings. In the current study, we aimed to investigate the impact of intrauterine insemination of hCG one day prior to fresh BT on the IVF outcomes. Our results did not show any additional benefit of this treatment regardless of the hCG used dosage. Indeed, the clinical pregnancy rate and the implantation rate were similar in all study groups with respectively 24.1% and 14.9% in the 500 IU treated group; 27.1% and 17.9% in the 1000 IU treated-group and 27.9 % and 18.7% in the control group without hCG treatment. All previous reports on the blastocyst stage used the optimal dose of 500 IU as described by Mansour *et al*. [10], with the exception of the study by Mostajeran *et al*. who tested the dose of 700 IU of hCG [16]. Based on the existing finding, we have hypothesized that the common dose of hCG supplementation could be probably insufficient to cause an improvement of clinical outcomes at the blastocyst stage.

Therefore, our study was conceived to assess both usual and higher doses of intrauterine hCG. Our findings clearly highlighted that neither a hCG dose of 500 IU nor of a double dose equivalent to 1000 IU had a beneficial effect on implantation and pregnancy rates. This may suppose that increasing the quantity of a missing or deceased cofactor does not necessarily lead to improved outcomes. Our findings are in contradiction to previous studies related to cleavage stage ET cycles [10-14], reporting a positive effect of intrauterine hCG application on clinical outcomes; but in concordance with some reports on BT cycles [9,15]. In fact, the previous suggestion, that a beneficial effect of intrauterine hCG infusion depends on the development stage of the transferred embryo, was recently supported by a Cochrane systematic review which concluded that women undergoing cleavage stage ET receiving an intrauterine hCG dose ≥500 IU have improved clinical pregnancy and live birth rates. Contrarily, there is insufficient evidence to support the use of this treatment for BT [23]. An earlier study, published as a conference abstract, reported an improvement of implantation and pregnancy outcomes after the transfer of blastocysts derived from fresh donor oocytes in women receiving intrauterine hCG injection [25]. Similarly, Mostajeran *et al*. found that pregnancy rate, although not significantly improved, trended upward in patients given an intrauterine injection of 700 IU of hCG before BT compared to the control group [16]. More recently, Liu X *et al*. reported a significant improvement of clinical pregnancy rate (7.5% versus 25.17%), implantation rate (29.19% versus 19.4%), and live birth rate (26.97% versus 17.22%) in patients with recurrent implantation failure treated with 500 IU of hCG 3 days before frozen BT compared to placebo group [3]. Contrarily, Hong *et al*. in a randomized clinical trial, reported no significant difference between the hCG-treated group and control group in implantation rate and ongoing pregnancy rate after BT for both fresh IVF cycles and frozen-thawed embryo transfer (FET) cycles [15]. Similarly, Wirleitner and colleagues, in a large cohort controlled study, by administering IU hCG at two different time points, could not find any evidence for improvement of clinical outcomes, neither 2 days before nor 3 minutes before BT independently of embryo quality [9]. Interestingly, the positive outcomes were recorded in only FET cycles [3,16] or in transfer cycles of embryos derived from oocytes donors [25]. However, the only three published studies (of which one was published as a conference abstract), analyzing data related to BT in fresh IVF cycles only, did not show any substantial benefit from intrauterine hCG application [9,15,26].

These findings are entirely consistent with our results and let's suppose that the endometrium response to the intrauterine hCG supplementation may be dependent on the type of ET cycle (fresh or frozen-thawed), or more accurately, on the endometrial status. In fact, it was assumed that endometrial receptivity in fresh IVF-ET cycles is different from FET cycles. This has been attributed to the suboptimal endocrine environment accompanying COH during IVF cycles, which may induce deleterious changes resulting in refractory endometrium and impaired implantation window [27]. However, in FET cycles or in recipients of oocyte donation, endometrial preparation and timing are considered to be closer to physiologic [15]. An original study indicated that external stimulation of the LH/hCG receptors (LHCGR) or the associated adenylyl cyclase enzyme is altered by high dosages of exogenous hCG injection, as it is done during cycles of COH [28]. This finding may explain why endometrium appears to be less responsive to the local signal of hCG under COH conditions. On the other hand, in the present study, we investigated the clinical efficacy of intrauterine hCG treatment one day before ET, a time point that has never been tested in all previous studies. Until now, the optimal moment of hCG administration in BT remains unknown, since it has not yet been established what timing will better promote endometrial receptivity. Of note, the majority of previous studies conducted the intervention a few minutes before ET. A recent meta-analysis revealed that the subgroup of women treated with 500 IU hCG within 15 minutes before ET exhibited significantly higher clinical outcomes compared to others time-subgroups, regardless of ET stage [21]. However, several questions remain in regard to the ideal timing of hCG infusion during BT cycles. It is thought that hCG probably has less time to exert its effect on the endometrium prior to BT on day 5 when compared with cleavage stage ET. Therefore, to compensate for this short duration of hCG action and to boost the therapeutic effect at the blastocyst stage some researchers proposed an earlier intrauterine hCG application either 6 hours [25,29], 2 days [9] or 3 days [3] prior to ET. However, interesting research demonstrated that precocious or prolonged local endometrial exposure to hCG (3-5 days) mediated a down-regulation and internalization of its own receptor (LHCGR) in endometrial epithelial cells, making them unresponsive to secreted hCG by hatched blastocyst [30].

In the same logic of extending hCG endometrial exposure time, but without causing detrimental effects on endometrial receptivity, we proposed an intrauterine hCG supplementation 24 hours before ET. But, unfortunately, we failed to prove any beneficial effect of this intervention in pregnancy outcomes. That suppose that changing the timing of intrauterine hCG supplementation may induce the secretion of disparate mediators involved in the implantation process and interfere differently with the embryo-endometrial crosstalk. Finally, this randomized prospective study conducted in Tunisian infertile women demonstrates that intrauterine hCG administration, for the first time, on the day before BT has no noticeable effect on implantation and pregnancy outcomes. However, these results may only be applied to our study population and could not be transposed to different clinical settings or other patient subgroups. Indeed, the clinical characteristics of patients receiving intrauterine hCG treatment could be an important confounding factor affecting its efficacy. Some data suggest that younger patients (< 38 years old [14] or < 35 years old [3]) or those with repeated implantation failure [3] are more likely to benefit from this treatment. In the current investigation, the study population was composed of mainly young women (with a mean age of 32.85 ± 5.10 years). However, our cohort was marked by a great clinical heterogeneity linked to wide variation in patient's age (ranged between 20 et 43 years), history of IVF-ET cycles (first or multiple cycles, with or without recurrent implantation failure), etiology of infertility (male factor, tubal factor, ovulation disorders, low gradelow-gradeiosis, idiopathic) and the used COH protocols. All these factors can have a clinical impact limiting the intrauterine hCG effectiveness. Therefore, our study population may not be the ideal population to benefit from this therapy. Moreover, the reported results are limited to the power provided by the sample size which is relatively small.

## Conclusion

Our study suggests that intrauterine hCG infusion could not be a beneficial approach to enhance clinical outcomes at the blastocyst stage in fresh IVF-ET cycles. Thus, its routine application in clinical practice is not recommended. However, there is yet a lack of strong evidence to draw a solid conclusion, owing to the wide variations in experimental design and study population criteria limiting the comparative effectiveness. Further, large and well-designed studies would be required to identify the optimal dose and time of hCG supplementation and the selection criteria of patients that could better benefit from this therapy.

### What is known about this topic

- It was demonstrated that intrauterine hCG infusion in the perinidatory interval significantly enhanced pregnancy and implantation rates during cleavage-stage ET; however, studies focusing on blastocyst transfer and usually testing the dose of 500 IU of hCG have shown controversial results.

### What this study adds

- Our study have shown that neither 500 IU of hCG nor a higher dose of 1000 IU of hCG injected in the uterine cavity one day before ET can have a beneficial effect at blastocyst stage.
